# Insomnia and Its Temporal Association with Academic Performance among University Students: A Cross-Sectional Study

**DOI:** 10.1155/2017/2542367

**Published:** 2017-06-29

**Authors:** Yohannes Gebreegziabhere Haile, Sisay Mulugeta Alemu, Tesfa Dejenie Habtewold

**Affiliations:** ^1^Department of Nursing, Debre Berhan University, Debre Berhan, Ethiopia; ^2^Mental Health and Psychosocial Support Program, International Medical Corps, Dolo Ado, Ethiopia; ^3^Department of Epidemiology and Psychiatry, University of Groningen, Groningen, Netherlands

## Abstract

**Introduction:**

Studies show that 9.4% to 38.2% of university students are suffering from insomnia. However, research data in developing countries is limited. Thus, the aim of the study was to assess insomnia and its temporal association with academic performance.

**Methods and Materials:**

Institution based cross-sectional study was conducted with 388 students at Debre Berhan University. Data were collected at the nine colleges. Logistic and linear regression analysis was performed for modeling insomnia and academic performance with a *p* value threshold of 0.05, respectively. Data were entered using EPI-data version 3.1 and analyzed using SPSS version 20.

**Results:**

The prevalence of insomnia was 61.6%. Field of study (*p* value = 0.01), worshiping frequency (*p* value = 0.048), marital status (*p* value = 0.03), and common mental disorder (*p* value < 0.001) were identified associated factors of insomnia. There was no significant association between insomnia and academic performance (*p* value = 0.53, *β* = −0.04). Insomnia explained 1.2% (*r*^2^ = 0.012) of the difference in academic performance between students.

**Conclusions:**

Nearly 3 out of 5 students had insomnia. We recommended that universities would endorse sleep quality and mental health illness screening programs for students.

## 1. Introduction

Insomnia, the most common sleep disorder, is the perception or complaint of inadequate or poor-quality sleep because of one or more of the following conditions: difficulty in falling asleep, frequent waking up during the night with difficulty for returning to sleep, waking up too early in the morning, or unrefreshing sleep [1–3]. It is the most common sleep-related complaint reported in the primary care setting [4] and a major chronic disease with reported rates of chronicity ranging from 24% to 45.8% [5, 6].

Epidemiological studies conducted in the United States (US) [7] and Hong Kong [8] report approximately 70 million and 2.2 million people who complain from symptoms of insomnia, respectively. A systematic review of 7 studies by Jiang et al. [9] shows that the prevalence of insomnia among university students is ranging from 9.4% to 38.2%. Furthermore, 32.5% to 62.3% university students in Nigeria, Libya, and Egypt suffered from insomnia [10–12].

On the one hand, physiological factors, schooling, and work schedules affect sleep quality [13]. On the other hand, poor sleep quality affects the human cognitive functions, such as information processing, learning, and the integration of intellectual records [14]. Poor sleep quality highly correlated with poor academic performance and reduced learning ability to perform basic activities, such as solving a mathematical problem [15–18]. The other detrimental effect of poor sleep quality includes reduced memory [19], reduced cognitive ability [20], risk of suicide [21], mental problems [22, 23], driving accident [24], and poor sleep hygiene practices [25, 26].

Even though adults are recommended to sleep 7 to 9 hours per day, university students sleep less than normal sleeping hour [12, 17, 26–28]. The new social environment, busy class schedule, change in sleeping environment, experiencing noise at night, and participating in night party alter the sleep quality and cause academic failure as a result [7, 29, 30]. Other factors that significantly predict insomnia among the student were chronic illness [8, 11, 31–33], gender [7, 34], age [10, 33], marital status, batch, and poor living style [26], inappropriate physical condition in the dormitories, academic task load, and field of study [10, 29, 33], self-perception [33], and irregular sleep pattern, and childhood adversities [11].

Leisure time, timing of sleep and wakefulness, napping, and good living condition help to improve academic achievement [23, 27]. Students who sleep for a longer duration have higher academic performance compared to short sleepers [35]. In addition, high-performing students had considerably earlier bedtime and waking time compared to low performers [23]. Therefore, identifying, minimizing, and/or avoiding risk factors of insomnia perhaps reduce stress and increase academic performance [18, 23, 36, 37].

Given the high public burden of insomnia, investigating insomnia and its association with academic performance has a scientific value for improving sleep quality and reducing attrition rate. National research data yet to be sufficient and poor sleep quality symptoms and risk factors remain underreported among Ethiopian students. The aim of the study was to assess insomnia and its association with academic performance.

## 2. Methods and Materials

### 2.1. Population and Procedure

Institution based cross-sectional study was conducted at Debre Berhan University from March to April 2015. Undergraduate students who were enrolled in 2014/2015 full-time study, were capable of independent communication, and provided informed written consent were included. All students were selected by proportionate stratified random sampling method. First, strata were created using each discipline/college as a cluster. Second, the list of students was obtained from the academic record office. Third, based on the calculated sample size, the required number of students was allocated to each college proportional to the total number of students enrolled. Finally, simple random sampling method was used to reach the individual student.

### 2.2. Sample Size Determination

The sample size was determined using single population proportion formula considering the prevalence of insomnia 50%, margin of error 5%, and confidence level of 95%. After adjustment for 10% nonresponse rate, the final sample size was 422.

### 2.3. Instrument

Data were collected from nine disciplines using self-administered questionnaire. The questionnaire had five sections: section one, demographic characteristics; section two, Pittsburgh Sleep Quality Index (PSQI) scale; section three, substance use habit; section four, physical and psychological complaints; and section five, sleep hygiene practice. The questionnaire was developed based on international guideline indicators, previous studies report in Ethiopia, and clinical expertise of principal investigators (Yohannes Gebreegziabher Haile and Sisay Mulugeta Alemu). PSQI was developed to measure the 30-day sleep quality and disturbances retrospectively [38]. PSQI scale includes the seven core domains of sleep: subjective sleep quality, sleep latency, sleep duration, habitual sleep efficiency, sleep disturbances, use of sleep medications, and daytime dysfunction. A validation study on sleep quality conducted in Africa revealed that the PSQI scale sensitivity was 72% and specificity was 54.5% with a threshold of global sum score >5 [38]. Other psychometric properties of PSQI scale were published elsewhere [39].

### 2.4. Variables

Insomnia was the primary outcome variable. PSQI scale was used to assess insomnia [38]. First, 7 separate scores corresponding to the seven sleep domains were generated. Second, for each student a single global sum score which ranged from 0 to 21 was constructed using the 7-component score. Finally, the student was categorized as “insomniac” if the global PSQI sum score was >5 and coded as “1”; the student was categorized as “normal sleeper” if the global PSQI sum score was ≤5 and coded as “0.” Academic performance was the secondary outcome variable. Self-reported cumulative grade point average (CGPA) was used as a proxy measure of academic performance. The explanatory variables were demographic characteristics, substance use habit, physical and mental health status, and sleep hygiene practice.

Smoking is the lifetime inhalation of the smoke of burning tobacco of cigarettes with any frequency as reported by students [40]. Khat is a plant with a mildly narcotic substance and consumed by chewing the green leaf; in this study, a self-reported lifetime khat chewing in any frequency was considered [41]. Alcohol drinking was defined as students subjective report of weekly or monthly consumption of beer or any traditional alcohol drink, such as Tela, Areke, and Tej. Physical complaints were operationalized as any self-reported signs and symptoms of physical illness and previously diagnosed disease by a physician during the last 30 days. Common mental disorder was defined as students subjective experience of anxiety and depression symptoms such as fatigue, irritability, forgetfulness, difficulty in concentration, and somatic complaints [42]. It was assessed using the 10-item Kessler Psychological Distress Scale (K10) and diagnosis made if sum score ≥7 [42]. Suicidal thought also was defined as a subjective students report of ideas about how to kill oneself during the last 30 days [43]. Worshiping was defined as any reported religious practice performed by students irrespective of their religion [42].

### 2.5. Data Processing and Analysis

Before analysis, the data passed through a stringent quality control process and inconsistencies, outliers, and missing values were checked. After checking the patterns of missing data values, multiple imputations (5x) were done considering that the data values were missing at random; therefore, the analysis was performed on each dataset per imputation and parameter estimates were pooled according to Rubin's rule [44]. First, all explanatory variables and insomnia were fitted to bivariate logistic regression model step-by-step. Then, variables were included in the final multiple logistic regression model if *p* value threshold reached 0.25. Finally, independently associated risk factors of insomnia were identified. Linear regression analysis was applied to model academic performance. The strength of association was interpreted using odds ratio and regression coefficient (*β*) and decision on statistical significance was made at a *p* value threshold of 0.05. EPI-data version 3.1 was used for data entry, variable coding, and cleaning while SPSS version 20 was used for analysis. This study was adherent to the Strengthening the Reporting of Observational Studies in Epidemiology (STROBE) statement [45].

### 2.6. Ethics Approval and Consent to Participate

In order to conform the Declaration of Helsinki (1964) and Population Screening Act (WBO), Debre Berhan University, Institute of Health Science and Medicine ethical review board approved the study protocol. Participation was voluntary and data was collected anonymously after obtaining written consent from each student.

## 3. Results

### 3.1. Baseline Characteristics of Students

In total, 388 (91.9%) students completed the self-administered questionnaire and 78.4% were male. The main reasons for nonresponse were a lack of interest and shortage of time. The mean age of the students was 22.1 year (SD ± 2.12). As illustrated in Table 1, 44.1% of students were enrolled in engineering discipline, 29.6% were fifth year, 72.4% were Amhara, and 85.1% were Orthodox Christian. In addition, 4.6% were cigarette smokers, 25.8% drank alcohol less than once per month, and 38.9% had headache. More than half (54.4%) of students had no regular sleeping and waking time, 75.3% had physically good sleeping environment, and 58.5% reported stress related to assignment, test, and exam.

### 3.2. Prevalence and Risk Factors of Insomnia

The prevalence of insomnia was 61.6%. The mean Pittsburgh Sleep Quality Index (PSQI) scale global sum score was 6.72 (SD ± 3.06).

The bivariate logistic regression analysis model showed that marital status, alcohol drinking, headache, back pain, fever suicidal thought, and common mental disorder were statistically significant (*p* value ≤ 0.05) risk factors of insomnia (Table 2).

Following multiple logistic regression modeling, field of study, worshiping frequency, marital status, and common mental disorder were found to be associated with insomnia. Students who enrolled in humanity and social science discipline were 4.4 times more likely to be insomniac compared to those who enrolled in natural and computational science discipline (*p* value = 0.01, 95% CI = 1.43–13.69). Students who are either divorced or married were less likely (70%) insomniac compared to single students (*p* value = 0.03, 95% CI = 0.10–0.87). Students who never worshiped were 3.7 times more likely to be insomniac compared to those who worshiped daily (*p* value = 0.048, 95% CI = 1.01–13.57). Furthermore, students with common mental disorder were 3.5 times more likely to be insomniac compared to those without common mental disorders (*p* value < 0.001, 95% CI = 1.99–5.99) (Table 2).

### 3.3. Insomnia and Academic Performance

The mean cumulative grade point average (CGPA) was 3.11 (SD ± 0.42) with a maximum of 4.00 and a minimum of 1.73 points. The mean CGPA of insomniac students was lower by 0.04 compared to normal sleepers, but not statistically significant (*p* value = 0.53, 95% CI = −0.19–0.10). Insomnia explained 1.2% (*r*^2^ = 0.012) of the difference in academic performance between students.

## 4. Discussion

In this study, 61.6% of students fulfilled insomnia diagnostic criteria, as defined by Pittsburgh Sleep Quality Index (PSQI) score >5. The associated factors were field of study, marital status, worshiping frequency, and common mental disorder. Further, there was no significant association between insomnia and academic performance.

The prevalence of insomnia in the present study was in congruence with cross-sectional studies report in Egypt [10] and Libya [12], but, on the other hand, higher than the prevalence in Nigeria (32.5%) [11] and Ethiopia [46, 47]. The possible explanations for this discrepancy were due to the difference in insomnia definition (use of different instrument), socioeconomic and cultural conditions, and sociodemographic characteristics, such as the age of students.

In this study, common mental disorder was significantly associated with insomnia; the odds of a student with common mental disorder to have insomnia were 3.5 times the odds of healthy student. This result was consistent with other studies conducted in Hong Kong, US, Brazil, and Italy [8, 31–33]. Another associated factor was marital status, where students who were either divorced or married less likely (70%) to have insomnia compared to single students. This finding was in line with the study done on US medical students [26].

We also found field of study was an associated risk factor of insomnia; the odds of humanity and social science students to have insomnia were 4.4 times the odds of natural and computational science. On the contrary, previous studies did not find any association [10, 29, 33]. Furthermore, the present study showed that students who never worshiped were 3.7 times more likely to be insomniac compared to those who worshiped daily. To the best of our knowledge, this finding was the first among students. The possible explanation was that worshiping helps to relieve stress and perhaps students sleep sufficiently [42].

Unlike the present study, previous researchers identified a significant association between insomnia and physical illness [11], gender [7, 34], age, inappropriate dormitory physical condition and dealing with the academic tasks [10, 33], self-perception of poor quality of life [33], academic demand (e.g., assignments), disturbing noise around sleeping area and participating in night parties [7, 29, 30], and family-related adverse childhood experience [11]. This difference may be attributed to the difference in the study population and small sample size.

Finally, the effect of insomnia on academic performance was examined. The mean cumulative grade point average (CGPA) of insomniac students was lower by 0.04 compared to normal sleepers, but not statistically significant. Insomnia explained 1.2% of the variability of students' CGPA. However, the study by Pagel and Kwiatkowski [16], Veldi et al. [48], and Medeiros et al. [17] concluded that insomnia significantly reduces academic performance. This discrepancy might be due to the difference in study population age, educational level, and operationalization of insomnia or poor sleep habit. In addition, this study measured only 30-day students' sleep quality despite the use of CGPA of all previous semesters or years. The other possible reason might be that majority of the students reported a CGPA of greater than 3.00, which might increase the risk of bias.

Sufficient sleeping hours facilitate the amalgamation of newly acquired knowledge with the existing one and improves memorization capacity [49]. Failure to get enough sleep is a challenge for students and causes daytime sleepiness, reduced memory and cognitive ability, decreased work efficiency, learning disability, and academic failure [17, 19, 20]. Researches on sleeping habit of university students are scarce in developing countries [10] as well as Ethiopia [46, 47]. As a result, evidence-based mental health care intervention has not been satisfactory. Thus, it is worthwhile to investigate the magnitude of sleep quality and disturbances using a standardized instrument. The result of this study would be useful for psychologist/psychiatrist to develop evidence-based prevention and therapeutic strategies for the students who are at risk/with insomnia. This study also provides further evidence for researchers, health planners, and policy makers. Furthermore, the finding of this study can be used as a baseline for future researchers.

This study attempted to recruit students from more than nine disciplines unlike most previous studies that focused only on one discipline (i.e., health science). In addition, a standardized PSQI scale was used to assess sleep quality and disturbances during the last 30 days retrospectively. However, this study has several limitations. First, self-administered data collection was used that might add recall and social desirability biases. Second, the cross-sectional nature of the study does not allow attribution of causality. Third, this study uses a self-reported CGPA to assess academic performance, which might not exactly and directly address the academic performance. Finally, the study was conducted only in one institution that might limit its external validity; however, this limitation is hampered by including students with different socioeconomic status and ethnic group.

## 5. Conclusion

To conclude, nearly 3 out of 5 students had insomnia. The risk factors were field of study, marital status, worshiping frequency, and common mental disorder. We recommended that universities would endorse sleep quality and mental health illness screening programs for students. Moreover, conducting interventional and longitudinal study will be helpful to advance understanding of the nature of insomnia. Lastly, we recommended further studies with larger sample size, diverse source population, variety of means of measuring academic performance, and different study design, to overcome majority of the limitations of this study.

## Figures and Tables

**Table 1:** Baseline characteristics of students.

Baseline characteristics	*n* = 388
*Demographic data*	
Sex, male *n* (%)	304 (78.4)
Discipline (field of study) *n* (%)	
Engineering	171 (44.1)
Natural and computational science	51 (13.1)
Agricultural science	24 (6.2)
Business and economics	59 (15.2)
Computer science and IT	25 (6.4)
Humanity and social science	36 (9.3)
Others^*∗*^	22 (5.7)
Batch *n* (%)	
1st year	89 (22.9)
2nd year	100 (25.8)
3rd year	70 (18.0)
4th year	14 (3.6)
5th year	115 (29.6)
Ethnicity, Amhara *n* (%)	281 (72.4)
Religion, Orthodox Christian *n* (%)	330 (85.1)
Worshiping, daily *n* (%)	171 (44.1)
Marital status, single *n* (%)	297 (76.5)
*Substance use habit and physical and mental health status*	
Cigarette smoking *n* (%)	18 (4.6)
Khat chewing *n* (%)	30 (7.7)
Drinking alcohol, less than once per month *n* (%)	100 (25.8)
Headache *n* (%)	151 (38.9)
Back pain *n* (%)	72 (18.6)
Fever *n* (%)	138 (36.3)
Suicidal thought *n* (%)	22 (5.7)
Common mental disorder *n* (%)	245 (63.1)
*Sleep hygiene practice*	
Regular sleeping and waking time *n* (%)	177 (45.6)
Good sleep environment (physically) *n* (%)	291 (75.0)
No noise around sleeping place *n* (%)	183 (47.2)
Attended night party *n* (%)	43 (11.1)
Stress related to exam, assignment, test *n* (%)	227 (58.5)
Exercise within 15 minutes of sleeping *n* (%)	51 (13.1)
Exercise before 3 hours of sleeping *n* (%)	61 (15.7)
Nap during the afternoon	237 (61.1)
Drink stimulants (coffee and tea) during nighttime *n* (%)	151 (38.9)
Eat heavy meal close to sleeping *n* (%)	79 (20.4)
Nighttime ritual *n* (%)	142 (36.6)
Using the bed only for sleeping purpose *n* (%)	157 (40.5)
Eating, drinking, reading, or watching TV on bed *n* (%)	245 (63.1)

^*∗*^Law, health science, and medicine

**Table 2:** Risk factors of insomnia.

Variables (reference category)	Bivariate regression model		Multiple regression model	
	*p* value	OR (95% CI)	*p* value	OR (95% CI)
*Demographic data*				
Sex, female (male)	0.51	0.9 (0.52, 1.39)		
Field of study, humanity and social science (natural and computational science)	0.07	2.4 (0.94, 6.42)		
Batch, 1st year (5th year)	0.23	1.4 (0.80, 2.55)	0.42	0.7 (0.23, 1.85)
Ethnicity, Oromo (Amhara)	0.71	0.9 (0.46, 1.69)		
Religion, Protestant (Orthodox Christian)	0.72	0.9 (0.40, 1.87)		
Worshiping frequency, never (daily)	0.10	2.6 (0.83, 8.08)	0.05	3.7 (1.01, 13.57)
Marital status, divorce and married (single)	0.008	0.3 (0.13, 0.73)	0.03	0.3 (0.10, 0.87)
*Substance use habit and physical and mental health status*				
Cigarette smoking (no)	0.19	0.5 (0.19, 1.38)	0.98	0.9 (0.23, 4.16)
Khat chewing (no)	0.09	0.5 (0.23, 1.13)	0.19	0.45 (0.13, 1.51)
Drinking alcohol, less than once per month (never)	0.03	1.7 (1.04, 2.91)	0.69	1.2 (0.58, 2.26)
Headache (no)	<0.001	3.0 (1.92, 4.77)	0.07	1.7 (0.96, 3.12)
Back pain (no)	0.003	2.6 (1.38, 4.82)	0.68	1.2 (0.53, 2.63)
Fever (no)	<0.001	2.5 (1.54, 3.89)	0.39	1.3 (0.72, 2.32)
Suicidal thought (no)	0.003	2.4 (1.35, 4.25)	0.78	1.2 (0.32, 4.51)
Common mental disorder (no)	<0.001	4.4 (2.80, 6.77)	<0.001	3.5 (1.99, 5.99)
*Sleep hygiene practice*				
Regular sleeping and waking time (no)	0.53	0.9 (0.58, 1.32)		
Sleep environment not good (physically) (yes)	0.05	1.6 (0.99, 2.71)	0.07	1.8 (0.95, 3.54)
No noise around sleeping place (yes)	0.03	0.6 (0.41, 0.95)	0.25	0.7 (0.42, 1.26)
Attended night party (no)	0.28	1.5 (0.73, 2.90)		
No stress related to exam, assignment, test (yes)	0.001	0.5 (0.31, 0.72)	0.31	0.8 (0.44, 1.30)
Exercise within 15 minutes of sleeping (no)	0.72	1.1 (0.60, 2.08)		
Exercise before 3 hours of sleeping (no)	0.20	1.5 (0.82, 2.64)	0.18	1.6 (0.80, 3.28)
No nap during the afternoon (yes)	0.94	1.0 (0.64, 1.51)		
Drink stimulants (coffee and tea) during nighttime (no)	0.78	1.1 (0.69, 1.64)		
Eat heavy meal close to sleeping (no)	0.21	1.4 (0.83, 2.40)	0.59	1.2 (0.62, 2.30)
Nighttime ritual (no)	0.45	1.2 (0.76, 1.83)		
Using the bed only for sleeping purpose (no)	0.54	1.1 (0.75, 1.74)		
No eating, drinking, reading, or watching TV on bed (yes)	0.32	0.8 (0.52, 1.23)		

## Abbreviations

DBU: Debre Berhan University
CMD: Common mental disorder
DSM IV TR: Diagnostic and Statistical Manual Fourth Edition Text Revision
GPA: Grade point average
ICD-10: International Classification of Mental and Behavioral Disorders
K 10: Kessler Psychological Distressing Scale
PSQI: Pittsburgh Sleep Quality Index
US: United States.

## References

[1] American Psychiatric Association (2000). *Diagnostic criteria from dsM-iV-tr*.

[2] American Sleep Disorders Association (1997). *International Classification of Sleep Disorders, Revised: Diagnostic and Coding Manual*.

[3] World Health Organization (1992). *The ICD-10 Classification of Mental and Behavioural Disorders: Clinical Descriptions and Diagnostic Guidelines*.

[4] Mahowald M., Kader G., Schenck C. (1997). Clinical categories of sleep disorders I. *Continuum*.

[5] Roberts R. E., Roberts C. R., Chan W. (2008). Persistence and change in symptoms of insomnia among adolescents. *Sleep*.

[6] Roberts R. E., Roberts C. R., Duong H. T. (2008). Chronic insomnia and its negative consequences for health and functioning of adolescents: a 12-month prospective study. *Journal of Adolescent Health*.

[7] Gaultney J. F. (2010). The prevalence of sleep disorders in college students: impact on academic performance. *Journal of American College Health*.

[8] Wong W. S., Fielding R. (2011). Prevalence of insomnia among Chinese adults in Hong Kong: a population-based study. *Journal of Sleep Research*.

[9] Jiang X.-L., Zheng X.-Y., Yang J. (2015). A systematic review of studies on the prevalence of Insomnia in university students. *Public Health*.

[10] Ibrahim J. M., Abouelezz N. F. (2011). Relationship between insomnia and computer use among students at Ain Shams University, Cairo, Egypt. *Egyptian Journal of Community Medicine*.

[11] James B. O., Omoaregba J. O., Igberase O. O. (2011). Prevalence and correlates of poor sleep quality among medical students at a Nigerian university. *Annals of Nigerian Medicine*.

[12] Taher Y. A., Samud A. M., Ratimy A. H., Seabe A. M. (2012). Sleep complaints and daytime sleepiness among pharmaceutical students in Tripoli. *Libyan Journal of Medicine*.

[13] Lima P. F., Medeiros A. L. D., Araujo J. F. (2002). Sleep-wake pattern of medical students: early versus late class starting time. *Brazilian Journal of Medical and Biological Research*.

[14] Gharagouzlo F., Nazari Z., Avakh A., Ebrahimi M. H., Poursadeghiyan M., Yarmohammadi H. (2016). Examining the prevalence of various sleep disorders indormitory living students in Kermanshah University of Medical Sciences-2014. *Journal of Current Research in Science*.

[15] Campos-Morales R. M., Valencia-Flores M., Castaño-Meneses A., Castañeda-Figueiras S., Martínez-Guerrero J. (2005). Sleepiness, performance and mood state in a group of Mexican undergraduate students. *Biological Rhythm Research*.

[16] Pagel J. F., Kwiatkowski C. F. (2010). Sleep complaints affecting school performance at different educational levels. *Frontiers in Neurology*.

[17] Medeiros A. L. D., Mendes D. B. F., Lima P. F., Araujo J. F. (2001). The relationships between sleep-wake cycle and academic performance in medical students. *Biological Rhythm Research*.

[18] Rodrigues R. N. D., Viegas C. A. A., Abreu E Silva A. A. A., Tavares P. (2002). Daytime sleepiness and academic performance in medical students. *Arquivos de Neuro-Psiquiatria*.

[19] Ram S., Seirawan H., Kumar S. K. S., Clark G. T. (2010). Prevalence and impact of sleep disorders and sleep habits in the United States. *Sleep and Breathing*.

[20] Pilcher J. J., Walters A. S. (1997). How sleep deprivation affects psychological variables related to college students' cognitive performance. *Journal of American College Health*.

[21] Hall R. C. W., Platt D. E., Hall R. C. W. (1999). Suicide risk assessment: A review of risk factors for suicide in 100 patients who made severe suicide attempts: evaluation of suicide risk in a time of managed care. *Psychosomatics*.

[22] Jewett M. E., Dijk D.-J., Kronauer R. E., Dinges D. F. (1999). Dose-response relationship between sleep duration and human psychomotor vigilance and subjective alertness. *Sleep*.

[23] Eliasson A. H., Lettieri C. J. (2010). Early to bed, early to rise! sleep habits and academic performance in college students. *Sleep and Breathing*.

[24] Pizza F., Contardi S., Antognini A. B. (2010). Sleep quality and motor vehicle crashes in adolescents. *Journal of Clinical Sleep Medicine*.

[25] Jefferson C. D., Drake C. L., Scofield H. M. (2005). Sleep hygiene practices in a population-based sample of insomniacs. *Sleep*.

[26] Brick C. A., Seely D. L., Palermo T. M. (2010). Association between sleep hygiene and sleep quality in medical students. *Behavioral Sleep Medicine*.

[27] Sweileh W. M., Ali I. A., Sawalha A. F., Abu-Taha A. S., Zyoud S. H., Al-Jabi S. W. (2011). Sleep habits and sleep problems among Palestinian students. *Child and Adolescent Psychiatry and Mental Health*.

[28] Oluwole O. (2010). Sleep habits in Nigerian undergraduates. *Acta Neurologica Scandinavica*.

[29] Eslami Akbar R. (2012). The prevalence of sleep disorder and its causes and effects on students residing in Jahrom University of Medical Sciences dormitories, 2008. *Journal of Jahrom University of Medical Sciences*.

[30] Ogbolu R. E., Aina O. F., Famuyiwa O. O., Erinfolami A. R. (2012). A study of insomnia among psychiatric out-patients in Lagos Nigeria. *Journal of Sleep Disorders & Therapy*.

[31] Sing C. Y., Wong W. S. (2011). Prevalence of insomnia and its psychosocial correlates among college students in Hong Kong. *Journal of American College Health*.

[32] Taylor D. J., Gardner C. E., Bramoweth A. D. (2011). Insomnia and mental health in college students. *Behavioral Sleep Medicine*.

[33] Angelone A. M., Mattei A., Sbarbati M., Di Orio F. (2011). Prevalence and correlates for self-reported sleep problems among nursing students. *Journal of Preventive Medicine and Hygiene*.

[34] Abdulghani H. M., Alrowais N. A., Bin-Saad N. S., Al-Subaie N. M., Haji A. M. A., Alhaqwi A. I. (2012). Sleep disorder among medical students: relationship to their academic performance. *Medical Teacher*.

[35] Kelly W. E., Kelly K. E., Clanton R. C. (2001). The relationship between sleep length and grade-point average among college students. *College Student Journal*.

[36] Parkerson Jr. G. R., Broadhead W. E., Tse C. J. (1990). The health status and life satisfaction of first-year medical students. *Academic Medicine*.

[37] Brown F. C., Buboltz W. C., Soper B. (2002). Relationship of sleep hygiene awareness, sleep hygiene practices, and sleep quality in university students. *Behavioral Medicine*.

[38] Aloba O. O., Adewuya A. O., Ola B. A., Mapayi B. M. (2007). Validity of the pittsburgh sleep quality index (PSQI) among nigerian university students. *Sleep Medicine*.

[39] Smith M. T., Wegener S. T. (2003). Measures of sleep: the insomnia severity index, medical outcomes study (MOS) sleep scale, Pittsburgh sleep diary (PSD), and Pittsburgh sleep quality index (PSQI). *Arthritis Care & Research*.

[40] Leone A., Landini L., Leone A. (2010). What is tobacco smoke? Sociocultural dimensions of the association with Cardiovascular risk. *Current Pharmaceutical Design*.

[41] Haile D., Lakew Y. (2015). Khat chewing practice and associated factors among adults in Ethiopia: further analysis using the 2011 demographic and health survey. *PLoS ONE*.

[42] Haile Y. G., Alemu S. M., Habtewold T. D. (2017). Common mental disorder and its association with academic performance among Debre Berhan University students, Ethiopia. *International Journal of Mental Health Systems*.

[43] Nock M. K., Borges G., Bromet E. J., Cha C. B., Kessler R. C., Lee S. (2008). Suicide and suicidal behavior. *Epidemiologic Reviews*.

[44] Rubin D. B. (2004). *Multiple Imputation for Nonresponse in Surveys*.

[45] von Elm E., Altman D. G., Egger M., Pocock S. J., Gøtzsche P. C., Vandenbroucke J. P. (2007). The strengthening the reporting of observational studies in epidemiology (STROBE) statement: guidelines for reporting observational studies. *Preventive Medicine*.

[46] Lemma S., Gelaye B., Berhane Y., Worku A., Williams M. A. (2012). Sleep quality and its psychological correlates among university students in Ethiopia: a cross-sectional study. *BMC Psychiatry*.

[47] Lemma S., Patel S. V., Tarekegn Y. A. (2012). The epidemiology of sleep quality, sleep patterns, consumption of caffeinated beverages, and khat use among Ethiopian college students. *Sleep Disorders*.

[48] Veldi M., Aluoja A., Vasar V. (2005). Sleep quality and more common sleep-related problems in medical students. *Sleep Medicine*.

[49] Gray P., Bjorklund D. F. (2014). *Psychology*.

